# USING INTENSIVE REPEATED MEASUREMENT TO EXAMINE THE AFFECTIVE CONSEQUENCES OF NEGATIVE RELATIONSHIP EXPERIENCES

**DOI:** 10.1093/geroni/igad104.1326

**Published:** 2023-12-21

**Authors:** Dakota Witzel, Kelly Cichy

## Abstract

Across midlife and older adulthood, daily relational contexts provide a lens through which daily experiences shape health and well-being. How daily relational contexts inform health and well-being is complex, with buffering and exacerbating effects of the relationship context on well-being reported in past work. One mechanism through which daily relational experiences influence health and well-being is through changes in emotions. The current series of talks examine how daily relational experiences (interpersonal stress, marriage and loneliness, perceived social isolation, caregiving stress) influence emotional well-being using innovative intensive repeated measurement designs. First, using eight days of daily diary data from the National Study of Daily Experiences, Dakota Witzel will consider the protective effects of perceived control on affective reactions to interpersonal daily stressors on the same day and the following day. Second, using a pre-registered, 14-day ecological momentary assessment design, Jin Wen will discuss the potential implications of affective reactions to interpersonal daily stressors on subsequent sleep quality. Third, using 14 days of ecological momentary assessments from the Einstein Aging Study, Karina Van Bogart will explore the moderative influences of marriage and cognitive impairment on the effects of daily emotional loneliness on intrusive thoughts. Finally, using a 14-day daily diary design, Frank Puga will present on the implications of daily caregiver stress and perceived social isolation on perceived emotional control. Kelly Cichy will serve as discussant and summarize the theoretical and methodological contributions of these studies for enhanced understanding of the dynamic interplay between relational experiences and emotional well-being in daily life.

